# Causes of Death on Antiretroviral Therapy: A Post-Mortem Study from South Africa

**DOI:** 10.1371/journal.pone.0047542

**Published:** 2012-10-16

**Authors:** Emily B. Wong, Tanvier Omar, Gosetsemang J. Setlhako, Regina Osih, Charles Feldman, David M. Murdoch, Neil A. Martinson, David R. Bangsberg, W. D. F. Venter

**Affiliations:** 1 Wits Reproductive Health and HIV Institute, University of the Witwatersrand, Johannesburg, South Africa; 2 Division of Infectious Diseases, Massachusetts General Hospital, Boston, Massachusetts, United States of America; 3 KwaZulu-Natal Research Institute for Tuberculosis and HIV, University of KwaZulu-Natal, Durban, South Africa; 4 School of Pathology, Faculty of Health Sciences, University of the Witwatersrand, Johannesburg, South Africa; 5 Department of Internal Medicine, Charlotte Maxeke Johannesburg Academic Hospital and Faculty of Health Sciences, University of the Witwatersrand, Johannesburg, South Africa; 6 Department of Medicine, Duke University Medical Center, Durham, North Carolina, United States of America; 7 Perinatal HIV Research Unit, University of the Witwatersrand, Johannesburg, South Africa; 8 Johns Hopkins University School of Medicine, Baltimore, Maryland, United States of America; 9 Center for Global Health, Massachusetts General Hospital, Ragon Institute of MGH, MIT and Harvard, Harvard Medical School, Boston, Massachusetts, United States of America; London School of Hygiene and Tropical Medicine, United Kingdom

## Abstract

**Background:**

Mortality in the first months of antiretroviral therapy (ART) is a significant clinical problem in sub-Saharan Africa. To date, no post-mortem study has investigated the causes of mortality in these patients.

**Methods:**

HIV-positive adults who died as in-patients at a Johannesburg academic hospital underwent chart-review and ultrasound-guided needle autopsy for histological and microbiological examination of lung, liver, spleen, kidney, bone marrow, lymph node, skin and cerebrospinal fluid. A clinico-pathologic committee considered all available data and adjudicated immediate and contributing causes of death.

**Results:**

Thirty-nine adults were enrolled: 14 pre-ART, 15 early-ART (7–90 days), and 10 late-ART (>90 days). Needle sampling yielded adequate specimen in 100% of kidney, skin, heart and cerebrospinal fluid samples, 97% of livers and lungs, 92% of bone marrows, 87% of spleens and 68% of lymph nodes. Mycobacterial infections were implicated in 69% of deaths (26 of 27 of these due to *M. tuberculosis*), bacterial infections in 33%, fungal infections in 21%, neoplasm in 26%, and non-infectious organ failure in 26%. Immune reconstitution inflammatory syndrome (IRIS) was implicated in 73% of early-ART deaths. Post-mortem investigations revealed previously undiagnosed causes of death in 49% of cases. Multiple pathologies were common with 62% of subjects with mycobacterial infection also having at least one other infectious or neoplastic cause of death.

**Conclusions:**

Needle biopsy was efficient and yielded excellent pathology. The large majority of deaths in all three groups were caused by *M. tuberculosis* suggesting an urgent need for improved diagnosis and expedited treatment prior to and throughout the course of antiretroviral therapy. Complex, unrecognized co-morbidities pose an additional challenge.

## Introduction

As antiretroviral therapy (ART) has been scaled up in sub-Saharan Africa, attention has focused on the problem of high rates of mortality in the first months of ART. [1], [2] Even when controlling for the state of immunodeficiency and opportunistic infections, patients in low-income settings have been shown to have significantly higher rates of early mortality compared to patients in high-income settings. [3] The reasons for this excess of early mortality are poorly understood.[4]–[7] To date studies addressing the etiology of these deaths have utilized chart-review and verbal autopsy, both of which are highly discordant with the gold standard of pathological autopsy.[8]–[11] Few pathological studies of adult patients dying of HIV-related illnesses in developing countries have been done, and none of these have included patients being treated with ART.[12]–[23].

To better understand causes of death in this group, we conducted a prospective post-mortem study of adults on ART who died after being admitted to hospital in Johannesburg, South Africa. A control group consisted of patients with advanced AIDS who were eligible for ART but had not yet received it. We ascertained causes of death though needle autopsy, chart review, and consideration of each case at a standardized clinico-pathologic conference.

## Methods

### Ethics Statement

The study was approved by the Ethics Committee of the University of Witwatersrand and the Institutional Review Board of Vanderbilt University. Subjects were consented into the study by one of two methods. Competent and willing subjects provided written consent for enrollment in the event of their death. After the death of disoriented or unconscious subjects, the next of kin provided written consent for participation. All clinical investigations were conducted according to the principles expressed in the Declaration of Helsinki.

### Setting and Subject Eligibility

Data were prospectively collected at Charlotte Makexe Johannesburg Academic Hospital, a 1088-bed, public, tertiary hospital located in central Johannesburg with a free ART program of over 3000 patients. At the time of the study, South African national guidelines allowed for ART initiation in patients with CD4 cell count <200 cells/mm^3^. In 2009, the HIV prevalence rate for Johannesburg’s province was 11.3% of the adult population; the reporting rate for tuberculosis cases was 469.9 per 100,000 per annum. [24].

Eligibility criteria for this study were: age >18 years, HIV-positive, either on ART or eligible for ART, and death occurring on one of the medical wards. Exclusion criteria were pregnancy and a history of defaulting or restarting ART.

### Study Procedures

#### Record review of pre-mortem data

A chart review was undertaken for each enrolled subject. Variables collected included: history of HIV and ART, history of tuberculosis (TB) and treatment, other comorbidities and opportunistic infections, clinical features of the presenting illness, and all radiology and laboratory results from hospital admission until the time of death.

#### Needle autopsy

The needle autopsy method was chosen because it can identify a pathogen in a significant proportion of HIV deaths and is faster, cheaper, and more acceptable to families than conventional autopsy. [25]–[27] As soon as possible after death, investigators aspirated cerebrospinal fluid (CSF) and performed standardized needle biopsies of the lungs (focusing on areas of abnormality on pre-mortem chest x-ray), liver, spleen, kidneys, and bone marrow. Additional biopsies of the heart, skin, lymph nodes and any accessible masses were obtained when pre- or post-mortem abnormalities were present. An ultrasound was used to locate the kidneys, spleen and heart. Other organs were located by external anatomical landmarks. Half-centimeter incisions were made in the skin overlying each biopsy site and closed with single sutures to achieve minimal cosmetic disturbance. Solid organ biopsies were obtained with 14-gauge core biopsy needles, bone marrow trephines with 14-gauge Jamshidi needles, CSF aspirated with 18-gauge syringes, and skin biopsied with standard punch tools. Multiple core biopsies (2 to 4 per skin incision) were obtained from the solid organs (lung, liver, spleen and kidney); biopsy specimens were, on average, 1.6 mm in diameter and ranged from 4–20 mm in length. Needle autopsies were performed by two investigators (E.B.W. and G.J.S.).

#### Histologic investigations

Tissue cores from each organ were preserved in formalin, embedded in paraffin and stained with Hematoxylin-eosin, Ziehl-Neelsen (ZN) for acid fast organisms, and Brown-Hopps modified tissue gram stain. Lung cores were stained with Grocott’s Methanamine Silver for fungi. Cytomegalovirus immunoperoxidase staining was performed when characteristic viral inclusions were noted. Additional immunohistochemical and special stains were performed as needed. Cytological examination was performed on CSF. Tissue processing and stains were performed by the National Health Laboratory Service with all interpretation performed by the study pathologist (T.O.).

#### Microbiologic investigations

Tissue cores were obtained using sterile technique and transported for same day processing. Specimens were subjected to aerobic and anaerobic bacterial cultures (lung, spleen); fungal culture (lung, spleen and CSF); mycobacterial culture (lung, spleen, liver, bone marrow, lymph node and CSF) and cryptococcal antigen testing (CSF). Antibiotic sensitivities were performed for positive bacterial cultures. Mycobacterial cultures were performed using the Bactec MGIT system (Becton Dickinson, New Jersey, USA) followed by species identification using the GenoType Mycobacterium CM Assay (Hain Lifesciences, Nehren, Germany) at the Johannesburg Tuberculosis Reference Laboratory. Real-time PCR-based LightCycler Mycobacterium detection assay (Roche Diagnostics, Germany) was performed on lung, liver, lymph node and CSF; specimens with sufficient leftover volume were also tested in the Xpert MTB/RIF PCR (Cepheid, California, USA) assay. Details of tissue preparation for the PCR assays have been published separately. [28].

#### Clinico-pathologic conferences

A committee of experienced infectious disease, pulmonary and pathology specialists from South Africa and the United States (C.F., D.M., N.M., R.O., T.O., G.S., W.D.F.V., E.W.) met at three clinico-pathologic conferences to adjudicate the immediate and contributing causes of death for each subject. The immediate cause of death was defined as the disease or condition directly leading to death whereas the contributing causes of death included other diseases or conditions believed to have contributed to the fatal outcome. [29] The committee reviewed the clinical history, pre-mortem investigations, post-mortem microbiology and post-mortem histology of each subject. A pre-designated rule dictated the interpretation of post-mortem bacterial and fungal cultures: an organism was considered pathologic only if 1) the same organism was cultured in pre-mortem culture or if 2) the affected organ showed appropriate vital inflammation on histology.

#### Categorization of causes of death

Causes of death were categorized as bacterial, fungal, mycobacterial, viral (non-HIV), neoplastic and non-infectious organ failure. Immune Reconstitution Inflammatory Syndrome (IRIS) was defined as worsening of disease with signs of clinically significant inflammation within 90 days of ART initiation despite appropriate treatment. TB IRIS was further defined as either paradoxical (known TB diagnosis with initial improvement on anti-tubercular therapy and subsequent paradoxical worsening) or unmasking (diagnosis of inflammatory TB within 90 days of ART initiation in a patient previously free of TB) in accordance with published consensus definitions. [30] Causes of death were defined as unsuspected by clinicians if they were revealed solely by the post-mortem investigations. Subjects in whom a cause of death remained imprecisely understood due to limitations of the post-mortem technique (ie. lack of sampling of the gastrointestinal tract or brain) were also recorded.

### Analysis

Baseline characteristics and the proportion of deaths attributable to each category were calculated overall and in three pre-planned sub-groups: pre-ART (<7 days of ART at the time of death), early-ART mortality (7–90 days of ART at the time of death), and late-ART mortality (>90 days of ART at the time of death). One-way analysis of variance and Fisher’s exact test were used to determine association between baseline characteristics and categories of mortality in these three groups. The sample size was dictated by the available budget.

## Results

### Pre-mortem Characteristics

From January – December 2009, 39 HIV-infected adults were enrolled. Six (15%) consented themselves prior to death; 33 (85%) were consented by family members after death. Half were women, median age was 36 years (IQR 32–40) and median CD4 count was 50 cells/mm^3^ (IQR 27–154, Table 1). Median length of hospitalization was 5 days (IQR 2–13) and 3 subjects received intensive care. Thirteen (33%) had received a diagnosis of tuberculosis and were on anti-tuberculosis therapy at the time of admission. Fourteen (36%) were on sulfamethoxazole-trimethoprim prophylaxis at the time of admission. Thirty-five (90%) received broad-spectrum antibiotics (most commonly amoxicillin/clavulanic acid, ceftriaxone, or pipercillin/tazobactam) and 23 (59%) received steroids during hospitalization (most often initiated empirically due to concern for hypoaldrenalism, *P. jerovici* pneumonia or IRIS).

**Table 1:** Pre-mortem characteristics of the study population.

	All deaths	Pre-ART deaths	Early-ART deaths	Late-ART deaths	p-value
	n = 39	n = 14	n = 15	n = 10	
Female, n (%)	19 (49%)	5 (36%)	8 (53%)	6 (60%)	0.47
Age in years, median (IQR)	36 (32–40)	37.5 (33–40)	33 (30–39)	36 (32–44)	0.30
CD4 cells/mm^3a^, median (IQR)	50 (27–154)	49 (18–111)	61 (46–154)	43 (27–175)	0.50
Days in hospital, median (IQR)	5 (2–13)	8 (2–15)	3 (2–11)	3.5 (1–9)	0.48
Days of ART, median (IQR)			32 (16–50)	326 (148–531)	
On TB treatment at admission, n (%)	13 (33%)	3 (21%)	7 (47%)	3 (30%)	0.36
On sulfamethoxazole- trimethoprim prophylaxis at admission, n (%)	14 (36%)	6 (43%)	5 (33%)	3 (30%)	0.84
Received broad-spectrum antibiotics during hospitalization, n (%)	35 (90%)	14 (100%)	14 (93%)	7 (70%)	0.07
Received steroids during hospitalization, n (%)	23 (59%)	9 (64%)	9 (60%)	5 (50%)	0.85

Categorized by duration of antiretroviral therapy (ART) at the time of death. Pre-ART deaths occurred in subjects who were HIV-positive and eligible for ART but had not yet received it (CD4 cell count <200 cells/mm^3^) or those who had received <7 days of ART. Early ART deaths occurred between 7–90 days of ART. Late ART deaths occurred after >90 days of ART.

a CD4 count measured most recently prior to death.

Twelve subjects were ART naïve and 2 had received <7 days of ART at the time of death; in this pre-ART group the median CD4 cell count was 49 cells/mm^3^ (IQR 18–111). Twenty-five subjects received >7 days of ART at the time of death; 15 were categorized as early-ART mortality with median duration of ART 32 days (IQR 16–50) and median CD4 cell count 61 cells/mm^3^ (IQR 46–154) and 10 were categorized as late-ART mortality with median duration of ART 326 days (IQR 148–531) and median CD4 cell count 43 cells/mm^3^ (IQR 27–175). Reported CD4 cell counts were those measured most proximally to death. Details of the antiretroviral regimen were available for 21 subjects, all of whom were on two nucleoside reverse transcriptase-inhibitors (11 stavudine/lamivudine, 5 didanosine/lamivudine, 5 tenofovir/lamivudine) and one non-nucleoside reverse transcriptase inhibitor (19 efavirenz, 2 nevirapine).

### Performance of the Needle Autopsy and Microbiologic Results

Median time from death to needle autopsy was 25 hours (range 5–55). The quality of each sample was assessed at histological examination and categorized as adequate (containing representative target tissue) or missed (Table 2). Adequate samples were obtained from the majority of biopsies: kidney (100%), skin (100%), heart (100%), CSF (100%), lung (97%), liver (97%), bone marrow (92%), spleen (87%), lymph node (68%). Bacterial cultures of the lung were pathogenic in 26% and contaminated in 33% of samples; in the spleen bacterial culture was pathogenic in 18% and contaminated in 15%. Pathogenic bacteria cultured from lung and spleen were *E. coli* (isolated in 8 cultures), *Acinetobacter* sp. (3), *Enterobacter* sp. (3), *Klebsiella* sp. (3), *Clostridium* sp. (2), *Proteus* sp. (2), and *Salmonella* sp.(1). Mycobacterial cultures were positive in 24% of livers, 23% of spleen, 19% of lung, 16% of lymph node, 15% of bone marrow and 10% of CSF samples. Mycobacterial cultures were positive in 16 subjects; with the organism identified as *M. tuberculosis* in 13, *M. avium* in 1, and unable to be further speciated due to contamination in 2. *M. tuberculosis* was identified by PCR in the tissues of 5 additional subjects. Fungal cultures had low rates of positivity: 8% in CSF, 8% in spleen and 3% in lung. The pathogenic fungi cultured from CSF, spleen and lung were *C. neoformans* (isolated in 6 cultures) and *C. albicans* (1).

**Table 2:** The yield of needle autopsy by site and investigation.

Site	Lung	Liver	Spleen	Kidney	Bone Marrow	Lymph Node	Skin	Heart	CSF
Method of sampling	blind core	blind core	ultrasound core	ultrasound core	trephine	blind core	punch	ultrasound core	aspiration
Attempted samples, n (adequate for analysis, missed)	39 (38, 1)	39 (38, 1)	39 (34, 5)	39 (39, 0)	39 (36, 3)	19^a^ (14, 5)	20^b^ (20, 0)	3^c^ (3, 0)	39 (39, 0)
Bacterial culture, n (positive, negative, contaminated)	39 (10, 16, 13)		39 (6, 26, 7)						
Fungal culture, n (positive, negative, contaminated)	37 (1, 25, 12)		39 (3, 33, 3)						37(3, 33, 1)
Mycobacterial culture, n (positive, negative, contaminated)	36 (7, 21, 8)	38 (9, 24, 5)	39 (9, 26, 4)		39 (6, 36, 7)	19 (3, 13, 3)			39 (4, 32, 3)
Cryptococcal antigen, n (positive, negative)									27 (3, 24)
Cytology, n (positive for neoplasm, negative)									30 (0, 30)

a Lymph nodes were biopsied in patients with palpable lymphadenopathy on post-mortem exam.

b Skin biopsy was performed in patients with rash on post-mortem exam.

c Heart was biopsied if pre-mortem history was suggestive of cardiac cause of death.

### Causes of Death: as Determined by the Clinico-pathologic Committee

Mycobacterial infection, confirmed microbiologically (in 21 cases) and/or histologically (in 26 cases), was the leading cause of death in the pre-ART (57%, 8/14) and on-ART groups (76%, 19/25) and contributed to the large majority of early-ART deaths (87%, 13/15, Table 3). Of the 27 cases of mycobacterial infection, 25 cases were determined to be due to *M. tuberculosis*, 1 to *M. avium*; and 1 to a mycobacterial infection that could not be specified. All mycobacterial deaths had evidence of disseminated infection (microbiological and histologic evidence detailed in Table 4). In those in whom mycobacterial infection caused death, it was the immediate cause of death in 56% (15/27). Of the 26 subjects with non-*M. avium* mycobacterial infection, 10 (37%) had been treated with TB therapy prior to admission the hospital; 7 (27%) were started on TB therapy during hospitalization and 9 (33%) were never initiated on TB therapy. Disseminated mycobacterial infection was revealed as a previously unsuspected cause of death at post-mortem in 9 subjects (6 in the early-ART mortality group).

**Table 3:** Causes of death by category.

	All deaths	Pre-ART deaths	Early-ART deaths	Late-ART deaths	p-value
	n = 39	n = 14	n = 15	n = 10	
Causes of death by category^a^					
Mycobacterial, n (%)	27 (69%)	8 (57%)	13 (87%)	6 (60%)	0.16
Bacterial, n (%)	13 (33%)	5 (36%)	6 (40%)	2 (20%)	0.45
Fungal, n (%)	8 (21%)	3 (21%)	4 (27%)	1 (10%)	0.79
Viral (non-HIV), n (%)	3 (8%)	2 (14%)	0 (0%)	1 (10%)	0.35
Neoplasm, n (%)	10 (26%)	3 (21%)	3 (20%)	4 (40%)	0.58
Organ dysfunction^b^, n (%)	10 (26%)	1 (7%)	5 (33%)	4 (40%)	0.13
Immune reconstitution inflammatory syndrome, n (%)	11 (28%)		11 (73%)		
Unsuspected by clinicians at time of death^c^, n (%)	19 (49%)	7 (50%)	8 (53%)	4 (40%)	0.85
Not satisfactorily explained by post-mortem technique, n (%)	5 (13%)	4 (29%)	1 (7%)	0 (0%)	0.13

Categorized by duration of antiretroviral therapy (ART) at the time of death. Pre-ART deaths occurred in subjects who were HIV-positive and eligible for ART but had not yet received it (CD4 cell count <200 cells/mm^3^) or those who had received <7 days of ART. Early ART deaths occured between 7–90 days of ART. Late ART deaths occurred after >90 days of ART.

a All causes of death (immediate and contributing) are included and each subject may have multiple causes of death.

b Non-infectious organ dysfunction, ie. pulmonary embolus or end stage renal disease.

c At least one cause of death was revealed only through the post-mortem investigations.

**Table 4:** Immediate and contributing cause(s) of death with supporting clinical, microbiologic and histologic findings.

A. Patients never treated with ART or with <7 days of ART at time of death										
ID	Age Sex	CD4	Days of ART	Days of TBT	Days ill	Key clinical features	Positive microbiology	Key histologic abnormalities	Immediate cause(s) of death	Contributing cause(s) of death
E03	39M	30	0	201	10	dyspnea, infiltrates	none	PJP (lung)	PJP	none
E04	29F	175	0	2	14	fever, HSM, ileus, ascites	*K. pneumoniae (blood*∧*),* MTB *(lung*∧∼, *spleen*∼, *liver*∼, *BM*∼*)*	Nec. gran. inflam (liver capsule%, spleen)	*K. pneumoniae* sepsis	Dissem. MTB*, Abdominal infection#
E08	37F	49	0	0	30	vomiting, dyspnea, fever, confusion	*C. neoformans* (blood∧, lung∼, spleen∼, CSF∼)	Cryptococcosis (lung, kidney)	Dissem. cryptococcosis*	none
E10	33F	45	0	2	40	cough, LAD, abdominal pain, confusion, pancytopenia	*A. baumannii* (sputum∧, spleen∼), *E. cloacae* (blood∧, lung∼, urine∧), *E. coli* (blood∧, urine∧), MTB (LN&)	Bacterial PNA, Nec. gran. Inflam. (liver, kidney, pancreas, BM, LN%), Pylonephritis	Polymicrobial sepsis	Dissem. MTB, Acinetobacter PNA*, Abdominal infection#
E11	46F	18	0	1	10	HA, meningismus, pulm. infiltrates	MTB (BM&, lung∼&, liver∼&, LN&, spleen∼)	Nec. gran. inflam (BM%, kidney%, liver%, lung%, LN%, spleen%)	Dissem. MTB	None
E12	54M	111	0	112	13	fever, dyspnea, hemiparesis	*E. coli* (lung∼)	Aspiration bronchopneumonia, Residual gran. Inflam. (BM, spleen)	Aspiration pneumonia	CVA, Dissem. MTB
E15	33M	168	0	1	4	fever, meningismus, lymphoctyic CSF	none	Non-specific lymphoplasmacytic inflam. (kidney, liver, lung, LN)	Meningitis#	none
E21	29F	6	0	47	30	fever, cough, jaundice, ileus	MTB (BM&, CSF∼, liver∼&, lung& LN∼&, spleen∼)	CMV pneumonitis, Nec. gran. inflam. (BM, kidney%, liver, lung%, pleura%)	Dissem. MTB	CMV pneumonitis*
E22	38M	180	0	0	16	fever, cough, abdo pain, pancytopenia, sepsis	*S.enterica* serogroup D (blood∧, spleen∼), MTB (lung&, LN&)	Nec. gran. inflam. (BM, liver, lung, pleura, spleen)	*S. enterica* sepsis*	Dissem. MTB
E29	44M	8	0	2	11	hemiparesis, CN deficit, fever, diarrhea	*K. pneumoniae* (lung∼, spleen∼)	Bilateral organizing PNA, Ill-defined gran. inflam. (spleen)	*K. pneumoniae* sepsis	Multifocal brain lesions#
E30	36M	66	0	9	30	jaundice, weight loss, diarrhea, LAD, HSM	Hepatitis B (blood∧), MTB (blood∧, lung∼, LN∼&, spleen∼)	Active Hepatits B, cirrhosis, ATN, Nec. gran. inflam. (BM, lung%, liver, LN%, spleen)	Dissem. MTB	Hepatiits B with cirrhosis
E32	39M		0	0	35	axillary mass, diplopia, dyspnea	None	High grade diffuse large B-cell lymphoma (kidney, liver, lung, LN, spleen)	High grade diffuse large B-cell lymphoma	none
E35	40M	70	4	0	10	fever, dyspnea, KS skin and tongue	MTB (blood∧, BM∼&, liver∼&, spleen∼)	Kaposi Sarcoma (skin), Nec. gran. inflam. (liver, spleen%)	Dissem. MTB*	KS
E36	37M	9	5	7	47	weakness, fever, LAD, pancytopenia	None	Hodgkins Lymphoma (BM, liver, spleen), Invasisve aspergillosis (lung)	Invasive pulmonary aspergillosis*	Hodgkins Lymphoma
**B. Patients treated with 7–90 days of ART at time of death**										
E25	39F	48	9	0	3	fever, meningismus, seizure	*C. neoformans* (blood∧, CSF ∧∼, spleen∼)	Cryptococcosis (BM, kidney, liver, lung, pancreas)	Disseminated cryptococcocis*	none
E16	40M	61	12	207	6	confusion,bedbound, DIC	*Enterobacter sp.* (lung∼), *Klebsiella sp.* (lung∼)	Aspiration bronchopneumonia	Enterobacter and klebsiella sepsis	Aspriration PNA, Neurologic process#
E37	33M	99	15	0	5	GI bleeding, shock, uremia	MTB (liver∼)	Nec. gran. inflam. (liver, lung, spleen), ESRD, ATN	Renal failure with uremic bleeding	Dissem. MTB (unmasking IRIS)*
E38	32F	17	16	1	5	diarrhea, confusion, renal failure	*Acinetobacter sp*. (lung∼), MAC (lung∼, spleen∼)	Gran. inflam. (BM, liver, lung, spleen), Bacterial PNA	Dissem. MAC (IRIS)*	Acinetobacter PNA
E05	31F	77	17	4	15	fever, meningismus, sepsis	MTB (blood∧, BM∼, CSF∼&, liver∼, lung∼&, sputum∧), *C. neoformans* ag (CSF∧∼)	Nec. gran. inflam.(spleen%, liver%, BM)	Dissem. MTB (unmasking IRIS)	Cryptococcal meningitis (IRIS)
E01	39F	22	32	0	40	seizures, ICH, vol. overload	AFB (BM∼)	Diffuse alveolar damage, ESRD	Renal failure	Dissem. mycobacterial infection (unmasking IRIS)*, ICH
E18	18F	61	32	0	17	hemoptysis, renal failure, HSM, hilar LAD	MTB (blood∧, BM∼, liver∼&, lung&, spleen∼)	Nec. gran. inflam. (BM, kidneys%, liver, lungs, spleen), ESRD	Dissem. MTB (unmasking IRIS)*	Renal failure
E26	30M	114	32	132	21	fever, confusion, lacunar infarcts, abnormal CSF	*Acinetobacter sp.* (lung∼)	Bacterial PNA, Gran. inflam. (BM, kidney, liver, spleen), PJP	Dissem. MTB (paradoxical IRIS)	Acinetobacter PNA*, PJP*
E06	32F	249	34	45	9	dyspnea, abdo pain, HSM	*C. dificile* (stool∧), MTB (CSF∼)	KS (lung, skin), Residual gran. inflam. (spleen)	Pulmonary KS (IRIS)	Dissem. MTB, C. difficile colitis
E33	43M	156	34	156	5	fever, HA, confusion, PNA	*C. neoformans* (CSF∧∼)	Cryptococcocis (lung), Nec. gran. inflam. (BM, kidney, liver, lung, spleen)	Dissem. Cryptococcosis (IRIS), Dissem. MTB (paradoxical IRIS)	none
E23	21M	46	37	60	25	fever, HSM, hilar LAD	MTB (lung&)	Nec. gran. inflam. (liver, lung%, spleen)	Dissem. MTB (paradoxical IRIS)	Dilated cardiomyopathy
E28	28F	353	50	93	30	diarrhea, abdominal distention, ascites	*C. dificile* (stool∧), *E. coli* (lung∼, spleen∼)	ATN, Residual gran. inflam. (liver, lung, LN, spleen)	*C. dificile* colitis	Dissem. MTB
E24	33M	50	51	86	38	night sweats, cough, CN palsies, hydrocephalus	MTB (lung&, CSF∧∼)	Gran. inflam. (BM, kidney, liver, lung, pleura)	Dissem. MTB (paradoxical IRIS)	none
E20	34F	6	55	0	3	dyspnea, skin and palate KS	AFB (liver∼), *E. coli* (lung∼, spleen∼)	Bacterial PNA, Gran. inflam. (kidneys, lung), KS (lung, LN, skin)	Dissem. KS	Dissem. MTB*, E. coli PNA*
E13	57M	154	73	0	21	fever, LAD, skin and palate KS	MTB (lung∼, spleen∼)	Nec. gran. inflam. (kidney, liver, lung, LN, spleen), KS (LN, skin)	Dissem. MTB (unmasking IRIS)*	KS
**C. Patients treated with >90 days of ART at time of death**										
E07	28M	47	92	184	9	swollen leg, dyspnea	MTB (lung&, BM&)	Pulmonary infarct, Nec. gran. inflam. (liver, spleen)	Pulmonary embolus	Dissem. MTB
E19	36F	175	132	27	30	ascites, abdo pain, pleural effusion, LAD	*Clostridium sp.* (lung∼, spleen∼), *Enterobacter sp* (spleen∼), *E. faecium* (blood∧), *E. coli* (lung∼, spleen∼)	Nec. gran. inflam. (LN), KS (LN, spleen), Schistosomiasis (liver, lung), Viral hepatitis	Polymicrobial sepsis	Hepatitis B, KS, MTB adenitis, Schistosomiasis
E17	48F	27	148	238	9	massive cervical LAD, HSM	None	High grade diffuse large B-cell lymphoma (BM, kidney, liver, lung, LN) spleen)	High grade diffuse large B-cell lymphoma	none
E02	34F		216	0	21	fever, dyspnea, abdominal distention	MTB (lung∧∼&, liver∼&, BM∼)	Nec. gran. Inflam. (kidney, liver%, spleen%, BM%)	Dissem. MTB	none
E31	32M	760	282	1	7	dypsnea, cardiomegaly, pleural efussions	none	Myocyte hypertrophy, Pulmonary interstitial fibrosis, Biventricular cardiac failure	Cardiomyopathy with biventricular failure	none
E27	42F	2	370	4	8	fever, pancytopenia, meningismus, LAD	MTB (blood∧, CSF∼&, liver&, lung∼, LN∼&, pus∧)	Gran. inflam (BM, liver%, lung%, spleen%)	Dissem. MTB	none
E14	56M	14	405	20	50	jaundice, LAD, tender HSM	*C. albicans* (spleen∼)	Candida pyelonephritis, Poorly differentiated malignancy (lung, soft tissue, pleura)	Candida urosepsis*	Metastatic malignancy*#
E34	44M	187	531	0	9	Infected KS lesion, dehydration, confusion, anemia	*P. mirabilis* (blood∧, lung∼, spleen∼), MTB (lung&, liver&, spleen&)	Gran. inflam. (kidney, liver, lung, spleen), KS (lung, skin)	*P. mirabilis* sepsis	Dissem MTB*, Dissem. KS
E09	29F	28	753	6	7	HTN, vol. overload, confusion, HSM	none	ESRD, Nec. gran. Inflam. (BM, liver%, lung, spleen)	Dissem. MTB	Renal failure
E39	36F	43	1113	0	21	cough, HSM, cardiac failure	none	Cardiac failure, Polymorphic B-cell NHL (heart, kidney, lung, spleen)	Polymorphic B-cell NHL*	Cardiac failure

**Symbols**: ∧pre-mortem culture;

∼ post-mortem culture;

& detected by PCR;

% Ziehl-Neelson stain positive;

* unsuspected at time of death;

# not satisfactorily explained by post-mortem technique.

**Abbreviations**: **AFB** – acid fast bacilli; **ag** – antigen; **ART** – antiretroviral therapy; **ATN** – acute tuberular necrosis; **BM** – bone marrow; **CN** – cranial nerve; **CSF** – cerebrospinal fluid; **CMV** – cytomegalovirus, **CVA** – cerebrovascular accident; **DIC** – disseminated intravascular coagulation;**ESRD** – end stage renal disease; **GI** – gastrointestingal; **gran.** – granulmonatous; **HA** – headache; **HSM** – hepatosplenomegaly; **HTN** – hypertension; **ICH** – intracerebral hemorrhage; **inflam. –** inflammation; **IRIS –** immune reconstitution inflammatory syndrome; **KS –** Kaposi sarcoma; **LAD –** lymphadenopathy; **LN –** lymph node; **MAC –** Mycobacterium avium complex; **MTB –** Mycobacterium tuberculosis; **nec. –** necrotizing; **NHL** – non-Hodgkins lymhoma; **PCR –** polymerase chain reaction; **PJP –** Pneumocystis iroveci pneumonia; **PNA –** pneumonia; **pulm –** pulmonary; **TBT –** tuberculosis therapy; **vol. –** volume.

Bacterial infections were the second highest overall cause of death (33%, 13/39); with pneumonia and sepsis as the most common manifestations (6 and 7 cases, respectively) and two cases of *C. difficile* colitis. Pneumonia and sepsis were both most often due to gram-negative enterobacteriaceae (7 cases) with 3 cases of noscomial *Acinetobacter* sp. pneumonia. Fungal infections contributed to 21% (8/39) of deaths overall (4 *C. neoformans*, 2 *P. jiroveci*, 1 *C. albicans*, and 1 invasive aspergillosis). The rates of bacterial and fungal causes of death were comparable in the pre-ART group (36% and 21% respectively) and early-ART group (40% and 27%), with a trend towards lower rates in the late-ART group (20% and 10%). Viral infections (other than HIV) contributed to 8% (3/39) of overall deaths (2 Hepatitis B, 1 cytomegalovirus pneumonitis). Schistosomiasis contributed to 1 death. Neoplasm contributed to 21% (3/14) of pre-ART deaths, 20% (3/15) of early-ART deaths and 40% (4/10) of late ART deaths (6 Kaposi’s sarcoma, 2 large B-cell lymphomas, 1 Hodgkins lymphoma, 1 metastatic carcinoma of unknown primary). Non-infectious organ failure contributed to 26% (10/39) of deaths (4 renal failure, 3 cardiac failure, 2 neurologic, 1 pulmonary embolus). Causes of death that had not been suspected clinically were revealed by the post-mortem technique in half of the cases (49%, 19/39). Causes of death that the committee determined to be insufficiently explained by the post-mortem technique were identified in 13% (5/39–2 abdominal processes and 3 brain processes, Table 4).

Infectious and neoplastic causes of death were frequently concurrent. Of the 27 subjects with disseminated mycobacterial infection as a cause of death, 62% (17/39) had at least one other infectious or neoplastic cause of death: bacterial infection (6), viral infection (2), fungal infection (2) and neoplasm (2). Three subjects had concurrent neoplasm, bacterial and mycobacterial infections; 1 had concurrent bacterial, fungal and mycobacterial infections, and 1 had concurrent neoplasm, bacterial, viral, parasitic, and mycobacterial infections.

IRIS was implicated in 73% (11/15) of early-ART deaths. IRIS was attributed to mycobacterial infection in 8 cases (6 *M. tuberculosis*, 1 *M. avium*, 1 unspecified mycobacterium), Kaposi sarcoma in 1 case, and both *M. tuberculosis* and *C. neoformans* in 2 cases. The median CD4 cell count for all IRIS cases was 77 cells/mm^3^ (IQR 46–154); and median duration of ART was 32 days (IQR 17–37). Of the 8 TB IRIS cases, 4 were unmasking IRIS and 4 paradoxical IRIS. The unmasking TB IRIS cases were all characterized by necrotizing granulomatous inflammation in multiple organs and positive *M. tuberculosis* tissue cultures (Table 4). The paradoxical TB IRIS cases had a median duration of antituberculosis therapy of 109 days (IQR 80–144). Two paradoxical TB IRIS cases displayed non-necrotizing granulomatous inflammation (ZN negative) and 2 displayed necrotizing granulomatous inflammation (ZN positive). Three of 4 cases had negative TB cultures and the only positive TB culture in this group was from CSF.

## Discussion

To our knowledge this is the first study from any developing country to use post-mortem investigations to report the causes of mortality for HIV patients on ART. The needle autopsy protocol described here was efficient and resulted in high histologic and mycobacteriologic yield. Tuberculosis was the leading cause of death regardless of ART status and was particularly high in subjects dying in the first 3 months of ART, in whom 87% had disseminated mycobacterial infection as an immediate or contributing cause of death. Multiple concurrent pathologies were common; 62% of subjects dying from mycobacterial infection had at least one additional infectious or neoplastic cause of death. IRIS contributed to 73% of early-ART mortality. Post-mortem investigations revealed clinically unrecognized causes of death in half of the subjects.

The finding that tuberculosis is the leading overall cause of death is consistent with prior autopsy studies of HIV patients from sub-Saharan Africa and India in the pre-ART era. In a 2010 meta-analysis of all autopsy studies of HIV patients from sub-Saharan Africa over the last two decades, tuberculosis was considered a cause of death in 32–45% of 593 autopsied adults. [11], [13], [18], [20], [31] In a series of 236 HIV-positive, ART-naïve patients from Mumbai, tuberculosis was implicated in 63% of deaths. [17] Our finding that every case of tuberculosis was disseminated beyond the lungs supports the finding by Martinson *et al.* that in their largely HIV infected, ART-naïve subjects, 97% of those with tuberculosis had evidence of disseminated disease during complete autopsy. [12] Our data on the importance of tuberculosis also support the conclusions of prior studies of cause-specific mortality of HIV patients on ART in low-income settings which used non-pathologic evidence from chart reviews and verbal autopsies. Tuberculosis is consistently among the leading causes of death in these studies, implicated in 16–18% of deaths in studies from Haiti, Senegal and Uganda and in 19–44% of deaths from two South African cohorts.[8], [9], [32]–[35] The higher number of deaths attributed to tuberculosis in our study is likely explained by the contribution of post-mortem investigations which revealed that a third of microbiologically and/or histologically-proven tuberculosis infections were clinically unsuspected at the time of death.

**Figure 1:**
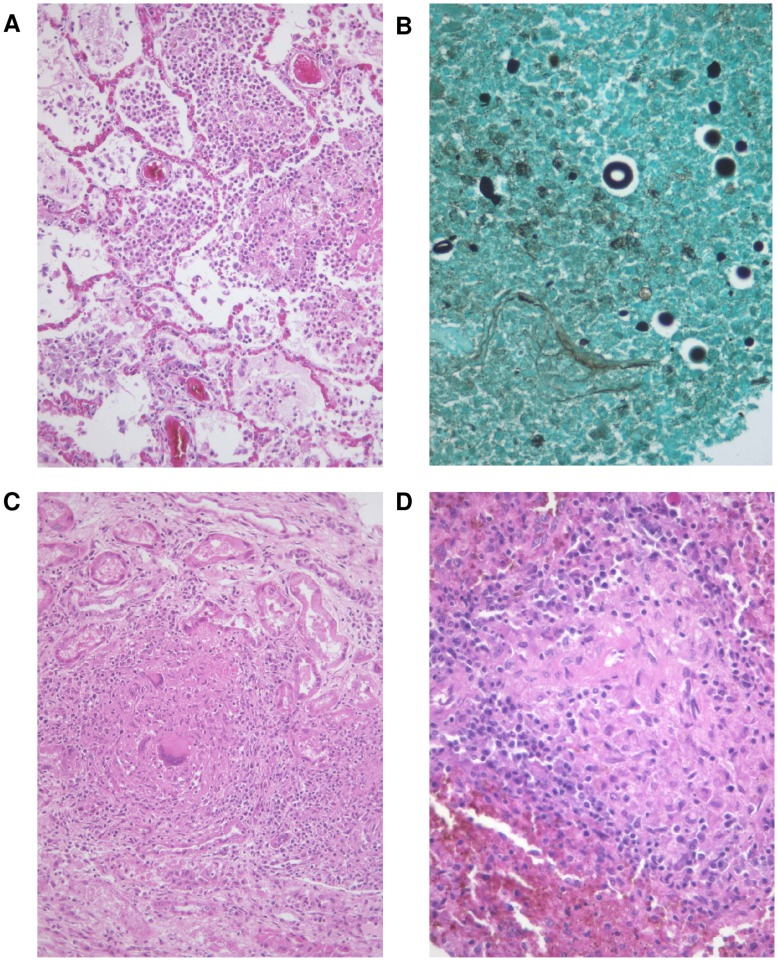
Simultaneous *C. neoformans* pneumonia and paradoxical *M. tuberculosis* Immune Reconstitution Inflammatory Syndrome (IRIS). At the time of death, this patient (E33) had been on anti-tuberculosis therapy for pulmonary tuberculosis for 5 months (with good response to treatment) and antiretroviral therapy for 1 month. Histologic sections demonstrate (a) suppurative consolidation of the lungs with (b) cryptococcal organisms apparent on Grocott’s Methanamine Silver (GMS) stain. Kidney (c) and spleen (d) demonstrate well formed necrotizing granulomatous inflammation, with negative Ziehl-Neelsen and GMS stains for organisms; these were thought to represent an exuberant inflammatory response due to paradoxical TB IRIS.

Our finding that IRIS contributed to over 70% of early-ART deaths highlights the importance and deadly potential of severe IRIS; in cohort studies based at ART clinics the condition has been described as usually self-limited and infrequently fatal. [36], [37] In cohort studies from Uganda and South Africa that have determined cause of death through chart-review and verbal autopsy, IRIS has been implicated in 7% of early-ART deaths and 17% of all ART-deaths respectively. [8], [33] Fatal IRIS has been reported, especially in central nervous system infections.[38]–[40] Although our study setting at a tertiary referral hospital may have influenced our findings, our high rate is supported by detailed clinicopathological data. The fatal cases described here challenge certain characteristics that have been used to define IRIS. In the consensus definition, the presence of another infection excludes IRIS [30]; however case E26 in this study demonstrates convincing evidence of paradoxical central nervous system TB IRIS with a simultaneous nosocomial bacterial pneumonia. In case E33, exuberant necrotizing granulomatous inflammation of multiple organs convinced the clinico-pathologic committee to diagnose concomitant paradoxical TB IRIS and unmasking cryptococcal IRIS (Figure 1).

This study does have a number of limitations. Small sample-size limited the ability of this study to find significant differences between pre-ART, early-ART and late-ART mortality and to pick-up low frequency causes of death. The pathology reported here is a minimum estimate as the needle autopsy was limited to sampled organs and likely missed important pathology in non-sampled organs (particularly in the abdomen/pelvis and cranium); similarly, areas of focal pathology within biopsied organs may have been missed. A study comparing results of needle and conventional autopsies found a concordance on primary cause of death in 67% of cases. [41] The predominance of gram-negative organisms in pre- and post-mortem cultures may be due to the translocation of enteric organisms across HIV-damaged gut mucosa and nosocomial pneumonias, but the striking lack of gram-positive cultures is most likely the result of pre-mortem broad-spectrum antibiotics and may have caused us to underestimate the contribution of pathogens like *S. aureus* and *S. pneumonia* to bacterial causes of death. Measuring HIV viral load at the time of death would have been helpful in determining adherence to and effectiveness of ART. This study may have overestimated the impact of IRIS due to miscategorization of overwhelming infections as unmasking IRIS; use of a prospective study design with serial measurements of CD4 cell count and HIV viral load might have more accurately made this distinction. We did not do mycobacterial drug-susceptibility testing which would also have helped clarify whether deteriorations after ART were due to IRIS or drug-resistant disease. [42], [43].The setting of our study in an urban tertiary referral hospital with access to advanced diagnostic procedures and intensive care facilities may limit the application of our findings to other settings.

The standardized needle autopsy described here was minimally mutilating, was efficient to perform, did not delay burial and had a very high pathological yield. While there is still a need for complete autopsies, in situations that preclude them a version of this needle autopsy protocol, perhaps modified to include brain and abdomen/pelvis sampling, has the potential to provide crucial post-mortem data.

Our findings demonstrate that tuberculosis is the major killer of HIV patients in sub-Saharan Africa, that it is frequently unrecognized and often accompanied by concurrent infections or neoplasms. Because simultaneous complex pathologies contribute to mortality, additional diagnoses should be sought for patients not clinically improving despite receiving treatment for known diagnoses. Additional study of the pathogenesis of and therapeutics for severe IRIS is needed. The development of a comprehensive response to the diagnosis and prevention of tuberculosis prior to and throughout the course of antiretroviral therapy is likely to have an enormous life-saving potential.
